# Spatial transcriptomic of hippocampal subfield‐specific pathology in Alzheimer’s disease and Primary Age‐Related Tauopathy (PART)

**DOI:** 10.1002/alz.095543

**Published:** 2025-01-09

**Authors:** Di Wu, Qilei Zhang, Anqi Liu, Yun Gong, Li Tianying, Zhengwu Xiao, Hongmei Xiao, Kuan‐Jui Su, Chuan Qiu, Hui Shen, Xiaoxin Yan, Hong‐Wen Deng

**Affiliations:** ^1^ Tulane University, New Orleans, LA USA; ^2^ School of Basic Medical Sciences, Central South University, Changsha, Hunan China; ^3^ School of Basic Medical Science, Central South University, Changsha, Hunan China

## Abstract

**Background:**

Alzheimer’s disease (AD) and primary age‐related tauopathy (PART) both hyperphosphorylated‐tau (pTau)‐positive neurofibrillary tangles (NFTs) but differ in the spatial pTau development and Aβ‐positive in the hippocampus. Cognitive status has been shown to be related to the overall hippocampal pTau burden, as well as the presence of β‐amyloid deposit. Spatially dissecting the subregions and the cell‐type‐specific molecular dynamics within the hippocampus among control, PART, and AD individuals is crucial for understanding the mechanisms underlying AD progression.

This study aims to construct the transcriptome‐wide, high‐resolution atlas of human hippocampus across control, PART, and AD subjects to delineate the cellular and molecular dynamics linked to the progression of AD.

**Methods:**

Human hippocampus samples were primary collected from six Asian subjects (two AD, three PART, and one control; aged 79‐92) for our 10X Visium spatial transcriptome and single‐nucleus RNA‐seq studies, as well as subsequent analysis. After identifying the subregions of hippocampus by clustering algorithms and manual annotation, differential gene expression (DGE) analysis was conducted to uncover the region‐specific molecular alternations across controls, PART, and ADs. Deconvolution using Bayesian methods provided a detailed assessment of cellular composition and transcriptional signatures. Cell‐Chat software was utilized to illustrate the regional specific inter‐cellular interactions.

**Results:**

Comparative analysis unveiled significant DEGs influencing specific hippocampal regions in control, PART, and AD samples, elucidating variations in cellular transcriptional expression levels across subject groups. Additionally, cell type‐specific gene communication pathways and directions are delineated, including the associated protein expressions and functional pathways.

**Conclusions:**

Our study of the human hippocampus reveals unique, cell‐type‐specific molecular alterations to PART and AD, compared to the control samples, illuminating potential mechanisms that drive the progression of AD.

